# A Proteinase 3 Contribution to Juvenile Idiopathic Arthritis-Associated Cartilage Damage

**DOI:** 10.3390/pathophysiology28030021

**Published:** 2021-06-23

**Authors:** Eric K. Patterson, Nicolas Vanin Moreno, Douglas D. Fraser, Gediminas Cepinskas, Takaya Iida, Roberta A. Berard

**Affiliations:** 1 Centre for Critical Illness Research, Lawson Health Research Institute, London, ON N6A 5W9, Canada; eric.patterson@lhsc.on.ca (E.K.P.); nvaninmoreno2021@meds.uwo.ca (N.V.M.); gcepinsk@uwo.ca (G.C.); takayaii@koto.kpu-m.ac.jp (T.I.); 2 Lawson Health Research Institute, Children’s Health Research Institute, London, ON N6A 5W9, Canada; douglas.fraser@lhsc.on.ca; 3 Department of Physiology and Pharmacology, Schulich School of Medicine and Dentistry, Western University, London, ON N6A 5C1, Canada; 4 Department of Paediatrics, Schulich School of Medicine and Dentistry, Western University, London, ON N6A 5C1, Canada; 5 Department of Medical Biophysics, Western University, London, ON N6A 5C1, Canada; 6 Division of Rheumatology, London Health Sciences Centre, Children’s Hospital, London, ON N6A 5W9, Canada

**Keywords:** proteinase 3, juvenile idiopathic arthritis, inflammation, synovial fluid, leukocyte elastase, myeloperoxidase

## Abstract

A full understanding of the molecular mechanisms implicated in the etiopathogenesis of juvenile idiopathic arthritis (JIA) is lacking. A critical role for leukocyte proteolytic activity (e.g., elastase and cathepsin G) has been proposed. While leukocyte elastase’s (HLE) role has been documented, the potential contribution of proteinase 3 (PR3), a serine protease present in abundance in neutrophils, has not been evaluated. In this study we investigated: (1) PR3 concentrations in the synovial fluid of JIA patients using ELISA and (2) the cartilage degradation potential of PR3 by measuring the hydrolysis of fluorescently labeled collagen II in vitro. In parallel, concentrations and collagen II hydrolysis by HLE were assessed. Additionally, the levels of the co-secreted primary granule protein myeloperoxidase (MPO) were assessed in synovial fluid of patients diagnosed with JIA. We report the following levels of analytes in JIA synovial fluid: PR3—114 ± 100 ng/mL (mean ± SD), HLE—1272 ± 1219 ng/mL, and MPO—1129 ± 1659 ng/mL, with a very strong correlation between the PR3 and HLE concentrations (r_s_ = 0.898, *p* < 1 × 10^–6^). Importantly, PR3 hydrolyzed fluorescently labeled collagen II as efficiently as HLE. Taken together, these novel findings suggest that PR3 (in addition to HLE) contributes to JIA-associated joint damage.

## 1. Introduction

Juvenile idiopathic arthritis (JIA) is an umbrella term used to describe a group of heterogenous conditions characterized by chronic arthritis of an unknown etiology, lasting more than six weeks, with onset before the 16th birthday [1]. The International League of Associations for Rheumatology’s classification criteria define seven homogenous, mutually exclusive subtypes of JIA: Systemic arthritis, oligoarthritis, rheumatoid factor (RF)-negative polyarthritis, RF-positive polyarthritis, psoriatic arthritis (PsA), enthesitis-related arthritis (ERA), and undifferentiated arthritis [1].

JIA is a chronic disease characterized by prolonged synovial inflammation that may cause structural joint damage. Short- and long-term morbidities are seen and may result from the disease or its treatments, which may impair the quality of life of patients and their families [2,3]. Despite the heterogeneity in clinical presentation and prognosis of the JIA subtypes, all share an autoimmune inflammatory process that inundates the joint with white blood cells, predominantly neutrophils [4,5].

Healthy synovial fluid is composed of lubricin, hyaluronic acid, and small molecules filtered from joint capsule capillaries [6,7]. However, in an inflamed joint, increases in the diameter and permeability of capillaries in the articular capsule allow the inundation of the joint with immune cells and large plasma proteins [8,9]. Neutrophils (PMNs) are the most abundant and the first immune cells to reach the joint. They carry intracellular granules containing large quantities of proteases such as proteinase 3 (PR3) and leukocyte elastase (HLE), as well as enzymes such as myeloperoxidase (MPO), used to destroy engulfed pathogens. However, during PMN degranulation or NETosis [10], these enzymes are released into the extracellular space where they can damage host tissue.

The presence of HLE in the synovial fluid and articular cartilage of rheumatoid arthritis (RA) patients and one JIA patient has been documented [11,12], along with its ability to degrade cartilage [13]. However, the role of the highly related and more recently isolated PR3 [14,15] has not been evaluated in JIA, where it likely contributes to the pathogenesis of the disease. Similarly to HLE, PR3 is a serine protease of approximately 30 kDa, capable of degrading a variety of extracellular matrix and cell–cell junctional proteins [14,16,17]. In addition to the primary granules, PR3 can also be constitutively expressed on the PMN outer membrane [18], and unlike HLE, PR3 can cleave the pro-group from IL-1β, turning it into its actively pro-inflammatory form [19].

We hypothesized that PR3 is abundant in the synovial fluid of patients with JIA and plays a contributing role to the joint damage associated with this disease. To this end, we measured the concentration of PR3 in the synovial fluid of patients with JIA and performed in vitro experiments to determine its ability to degrade collagen II relative to HLE.

## 2. Materials and Methods

### 2.1. Experimental Design

This study was approved by the ethics review board at Western University (London, ON, Canada). Informed consent was obtained from sequential patients with JIA and/or a parent or guardian at the Children’s Hospital, London Health Sciences Centre (London, ON, Canada), at the time of intra-articular steroid injection with triamcinolone hexacetonide. Eighteen patients, 15 females and three males, under the age of 18 were consented for this study between March and August 2016 (Table 1). There were no exclusion criteria. After collection, the patient synovial fluid was immediately centrifuged, aliquoted, and stored at −80 °C at the Translational Research Centre (London, ON, Canada) until analysis.

### 2.2. Enzyme-Linked Immunosorbent Assays (ELISA)

ELISA was performed for PR3 (Biomatik, Kitchener, Canada, EKU06958), HLE (Abcam, Toronto, Canada, ab119553), and MPO (Abcam, Toronto, Canada, ab119605), following the manufacturer’s protocol. Synovial samples were diluted in their respective ELISA kit’s sample diluents (PR3 1:100, HLE 1:100, or 1:800, MPO 1:100) and assayed in duplicate. The standards provided in the kit were assayed in triplicate.

### 2.3. Collagen II Hydrolysis by Serine Proteases

Mixtures were prepared on ice in a 96-well plate as follows: For each well, 25 µL of ~1 mg/mL Collagen II-FITC (Sigma, Oakville, ON, Canada, C4486) dissolved in 0.1 M acetic acid, was combined with 2.5 µL of 1 M NaOH and 175 µL of Hank’s balanced salt solution (HBSS) (pH 7.4, 100 mM HEPES buffer, no bicarbonate, no phenol red), mixed and incubated at room temperature in the dark for 4 h to overnight to allow fibril formation. After washing for 3 × 10 min with HBSS to remove unincorporated dye or collagen monomers, 200 µL of the indicated enzyme solution was added in triplicate wells and incubated for 2 h at 37 °C (enzyme solutions were either native PR3 (Athens Research, Athens, GA, USA, #16-14-161820) or HLE (Athens Research, Athens, GA, USA, #16-14-051200) diluted to 1 µg/mL in HBSS). Subsequently, 150 µL from each well was then transferred to a new black-walled 96-well plate (Grenier Bio-one, Monroe, LA, USA, #655096) and read in a Victor 3 plate reader (Perkin Elmer, Branford, CT, USA) with absorbance/emission of 485/520 nm.

### 2.4. Statistics

Statistical calculations were made using GraphPad Prism v9.1. Non-parametric tests were used, since the data were not normally distributed. Correlations between enzyme concentrations and cells in synovial fluid were calculated using Spearman’s rho. For correlation significance testing, Bonferroni’s correction was used to adjust alpha to 0.017 to account for multiple comparisons. The Kruskal–Wallis test with Dunn’s multiple comparison test was used to test for differences in collagen II hydrolysis, and the results were considered significant when *p* < 0.05.

## 3. Results

### 3.1. Patient and Clinical Data

The demographic information of the subjects from which synovial fluid was obtained is presented in Table 1. Fifteen of the 18 subjects were female, though since JIA occurs at roughly twice the rate in females compared to males [20,21], this is within expectations. The average age of patients was 9.8 ± 5.4 years (mean ± SD). Eleven subjects had oligoarthritis and seven polyarthritis RF-negative; 14/19 samples were collected from a right knee aspirate. In one patient, synovial fluid was sampled from both knees. Additionally, we found that the synovial WBC count in patients with oligoarthritis was lower than in polyarthritis (8.5 ± 2.4 × 10^6^/mL vs. 15.2 ± 14 × 10^6^/mL), though not statistically significant. Three patients had JIA-associated chronic anterior uveitis, but no other comorbidities were present in this cohort.

### 3.2. Synovial Fluid Enzyme ELISAs

**Figure 1:**
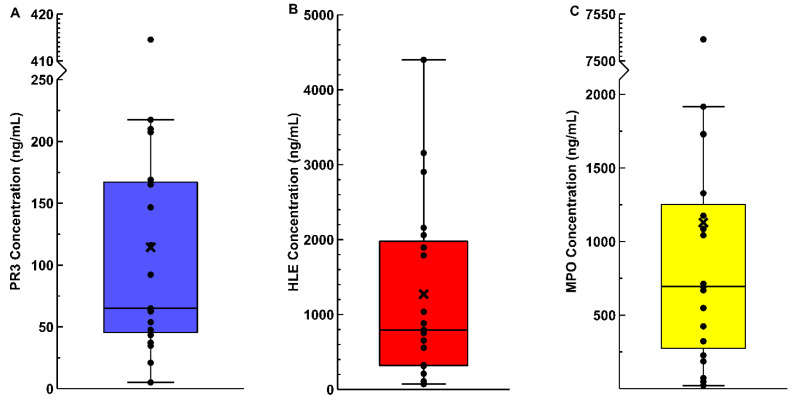
PMN enzyme synovial fluid concentrations. Samples were thawed on ice, diluted in their respective sample diluents, and their concentrations determined by ELISA. (**A**) Synovial fluid PR3 concentrations; (**B**) synovial fluid HLE concentrations; (**C**) synovial fluid MPO concentrations. Whiskers extend to points ≤1.5 × IQR, while means are represented by “×”.

We first measured synovial fluid PMN enzyme concentrations by ELISA (Figure 1). The PR3 concentrations (Figure 1A) were found to be 114 ± 100 ng/mL, while the HLE concentrations (Figure 1B) were 1272 ± 1219 ng/mL. The concentrations of MPO (Figure 1C) were found to be 1129 ± 1659 ng/mL. There was a strong correlation between the PR3 and HLE concentrations (r_s_ = 0.898, *p* < 1 × 10^−6^), the PR3 and MPO concentrations (r_s_ = 0.723, *p* < 0.001), and the HLE and MPO concentrations (r_s_ = 0.700, *p* = 0.001). The correlations between the various enzyme concentrations and synovial neutrophil counts were weak-to-moderate (r_s_ = 0.297–0.516), but non-significant.

### 3.3. PR3 and HLE Effects on Collagen II

**Figure 2:**
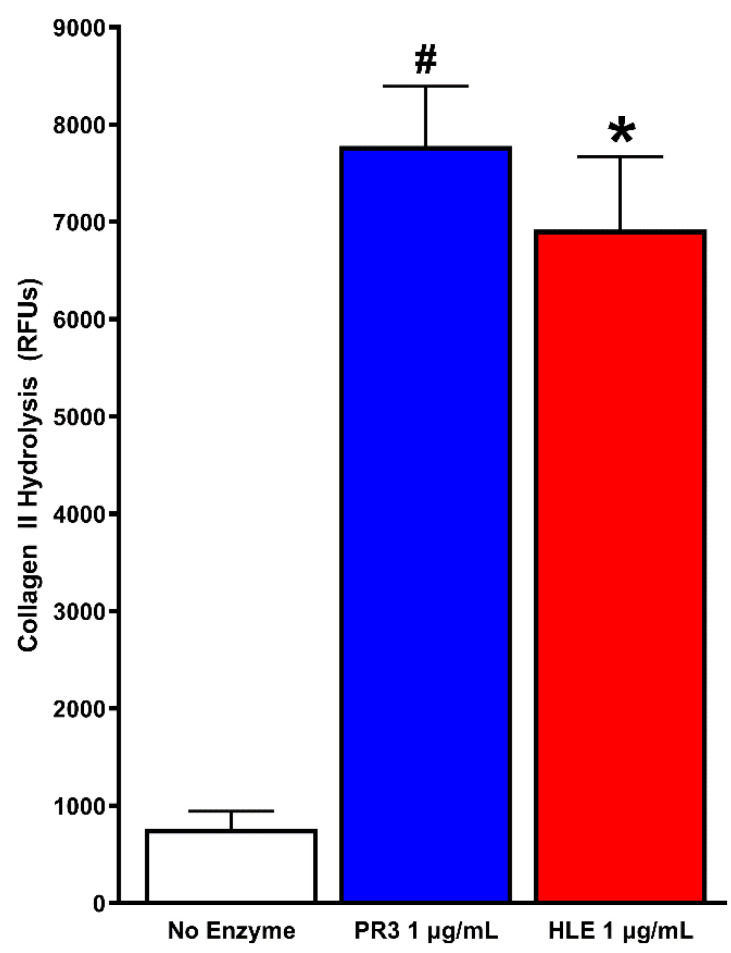
PR3 and HLE degrade collagen II. FITC-labeled collagen II fibrils were incubated with native PR3 or HLE in HBSS for 2 h at 37 °C. Samples were collected and centrifuged, and the amount of hydrolyzed collagen was measured by reading the supernatants in a Victor 3 plate reader at ex:485/em:520. *N* = 8 per group, # *p* < 0.001 vs. no enzyme and * *p* < 0.05 vs. no enzyme by the Kruskal–Wallis test.

Next, we assessed PR3 and HLE’s potential to damage cartilage by proteolytic degradation of collagen II (a major protein component of cartilage) in vitro. We used equal concentrations of both proteases (1 µg/mL) to more easily compare their effects. As shown in Figure 2, both PR3 and HLE significantly hydrolyzed FITC-labeled collagen II compared to no enzyme controls. Despite PR3 having a greater activity than HLE (7781 ± 1735 RFUs vs. 6920 ± 2134 RFUs), there was no statistically significant difference between the two enzymes.

## 4. Discussion

The current study demonstrated that PR3, an enzyme that has so far been largely overlooked in studies of proteolytic damage, is present in the synovial fluid of JIA patients. Furthermore, proteolysis of collagen II by PR3 suggests its potential role in contributing to protease-mediated joint damage in JIA.

A key characteristic of inflammatory arthritis diseases is inflammatory cell recruitment (e.g., PMN) to joints, leading to the accumulation of large amounts of HLE and MPO in synovial fluid, such as in JIA [22,23] and RA [11,24]. It has been long acknowledged that HLE is a major contributor to proteolytic articular joint injury [13]. To the best of our knowledge, this study is the first to report PR3 concentrations in synovial fluid. A previous study found PR3–A1AT (α1 anti-trypsin) complexes in RA synovial fluid, but did not report their concentrations, only correlations with HLE–A1AT complexes [25]. The synovial fluid HLE concentrations from our work (1272 ± 1219 ng/mL) were low compared to those reported in RA patients’ synovial fluid (5063 ± 1673 ng/mL [11]), though comparable to those in JIA in the 2000 ng/mL range [22] (the latter was calibrated against porcine pancreatic elastase).

Our finding of higher synovial WBC counts in polyarticular JIA compared to oligoarticular JIA, while not statistically significant, is consistent with other studies [26,27]. The weak correlations we observed between synovial fluid enzymes and synovial PMNs (their primary source) is likely due to the degranulation process. In order to be free in the synovial fluid, enzymes must first have been released by PMN degranulation or NETosis, and the remaining PMN debris is unlikely to be detected as a PMN.

While this study primarily concerned PR3 proteolysis, we also measured synovial fluid MPO concentrations. Although MPO causes joint damage through oxidative mechanisms, its activity also exacerbates proteolytic damage by oxidant-induced inactivation of A1AT and A2MG, the major native inhibitors of PR3 and HLE [28,29]. Our findings indicate high synovial fluid MPO concentrations in JIA patients, confirming PMN degranulation and suggesting a potential contribution by MPO to the pathophysiology of JIA.

One of the key findings of this study is that PR3 effectively degrades collagen II, a major component of the cartilage matrix. Our in vitro model of cartilage proteolysis used PR3 and HLE at concentrations similar to those measured for HLE in the synovial fluid. However, it is likely that these enzymes are present in an inflamed joint’s cartilage at concentrations even higher than in the synovial fluid, as reported previously [11,12], thus increasing their potential for damage. Our measured PR3 concentrations were approximately one tenth that of HLE; however, using an equal concentration of both enzymes in our proof-of-principle experiments permitted a more comparable evaluation of their proteolytic activity. It is of note that PMNs contain approximately two-fold more PR3 than HLE [30,31] and both enzymes are packaged in the same granules. As such, lower PR3 concentrations compared to HLE in the synovial fluid may result from PR3 being degraded or removed more quickly than HLE, or PR3 may also be trapped in NETs or cartilage matrix. However, the strong correlations observed between the three enzymes measured suggest they are co-released from PMNs, as would be expected.

Despite the lower PR3 concentration compared to HLE, there are critical factors that may increase PR3′s importance in the overall proteolytic damage to tissue. First, PR3 is more active than HLE at the lower pH found in inflamed tissues [14], and second, native protease inhibitors selectively inhibit HLE over PR3 [32]. Furthermore, neutral serine proteases bound to cartilage [33] or other surfaces [32,34,35] are resistant to inhibition by native large-molecule inhibitors such as A1AT and A2MG. Indeed, PR3, HLE, and MPO’s cationic charge facilitate their interaction with anionic membrane surfaces [31]. This tends to favor their retention on cartilage surfaces and effectively increase the concentrations experienced by these membrane surfaces.

Intra-articular injections with triamcinolone hexacetonide are a common treatment used in JIA, and taken with the importance of neutrophil proteases in joint damage, possible experimental treatment with localized protease inhibitors may provide a synergistic therapeutic approach, particularly in the refractory joint disease in JIA. Protease inhibitors may compliment corticosteroids by acting immediately on the proteases already present and those about to be released from PMNs in the cartilage and could potentially diminish the possible adverse effects that systemic inhibitor administration may have. Furthermore, this would allow for higher inhibitor concentrations than would be possible with systemic administration. We previously demonstrated that carbon monoxide-releasing molecules, a class of emerging anti-inflammatory compounds, inhibit MPO activity [36] and limit PMN migration across endothelial cells [37].

Despite these interesting findings, the current study has limitations. Most importantly, we lacked a healthy control group for synovial fluid measurements. Ethically, fluid aspirates from the small amounts present in a healthy joint, particularly from children, is not possible. Future work could compare JIA fluid to other transient inflammatory arthritis conditions in childhood such as transient synovitis. Second, while collagen II is a major component of cartilage, alone it does not fully replicate the complex collagen-based matrix and the extracellular milieu found in an inflamed joint.

In summary, this is the first study to present the concentrations and cartilage-degrading potential of PR3 (while confirming elevated levels of HLE and MPO, both markers of neutrophil recruitment and degranulation) in the synovial fluid of JIA patients, thus highlighting the previously overlooked contribution of PR3 to the pathophysiology of JIA.

## Figures and Tables

**Table 1:** Patient demographic information.

Age (Years)	Sex	JIA Diagnosis	Knee Aspirated	Synovial Fluid WBC (×10^6^/mL)	Synovial Fluid PMN (×10^6^/mL)
2	F	Polyarthritis RF-neg	Right	NA	NA
5	F	Oligoarthritis JIA	Right	13.26	8.36
6	F	Polyarthritis RF-neg JIA	Right	NA	NA
2	F	Psoriatic JIA	Right	25.66	22.33
7	F	Oligoarthritis JIA	Right	NA	NA
17	F	Oligoarthritis-extended JIA	Right	NA	NA
10	M	Oligoarthritis JIA	Right	NA	NA
8	F	Oligoarthritis JIA	Right	7.35	4.41
17	F	Polyarthritis RF-neg JIA	Left	6.22	3.36
3	F	Oligoarthritis JIA	Right	9.91	0.59
5	F	Oligoarthritis JIA	Right	9.53	3.43
			Left		
15	F	Polyarthritis RF-pos JIA	Right	28.56	24.56
14	F	Oligoarticular JIA	Right	8.51	4.08
14	M	Enthesitis-related JIA	Right	8.62	3.02
8	M	Oligoarthritis JIA	Left	0.48	0.08
13	F	Oligoarticular JIA	Right	5.08	1.27
17	F	Oligoarthritis JIA	Right	6.23	1.62
14	F	Oligoarthritis JIA	Left	7.57	1.06

Abbreviations: JIA, juvenile idiopathic arthritis; PMN, polymorphonuclear neutrophil; NA, not available; RF, rheumatoid factor; WBC, white blood cell.

## Data Availability

Non-identifying data are available upon reasonable request.
